# Healthcare facility-based strategies to improve tuberculosis testing and linkage to care in non-U.S.-born population in the United States: A systematic review

**DOI:** 10.1371/journal.pone.0223077

**Published:** 2019-09-30

**Authors:** Amanda P. Miller, Mohsen Malekinejad, Hacsi Horváth, Janet C. Blodgett, James G. Kahn, Suzanne M. Marks

**Affiliations:** 1 Philip R. Lee Institute for Health Policy Studies, University of California, San Francisco, San Francisco, California, United States of America; 2 Division of Infectious Disease and Global Public Health, School of Medicine, University of California, San Diego, La Jolla, California, United States of America; 3 Global Health Sciences, University of California, San Francisco, San Francisco, California, United States of America; 4 Division of Tuberculosis Elimination, National Center for HIV/AIDS, Viral Hepatitis, STD and TB Prevention, United States Centers for Disease Control and Prevention (CDC), Atlanta, Georgia, United States of America; University of Mississippi Medical Center, UNITED STATES

## Abstract

**Context:**

An estimated 21% of non-U.S.-born persons in the United States have a reactive tuberculin skin test (TST) and are at risk of progressing to TB disease. The effectiveness of strategies by healthcare facilities to improve targeted TB infection testing and linkage to care among this population is unclear.

**Evidence acquisition:**

Following Cochrane guidelines, we searched several sources to identify studies that assessed strategies directed at healthcare providers and/or non-U.S.–born patients in U.S. healthcare facilities.

**Evidence synthesis:**

Seven studies were eligible. In a randomized controlled trial (RCT), patients with reactive TST who received reminders for follow-up appointments were more likely to attend appointments (risk ratio, RR = 1.05, 95% confidence interval 1.00–1.10), but rates of return in a quasi-RCT study using patient reminders did not significantly differ between study arms (*P* = 0.520). Patient-provider language concordance in a retrospective cohort study did not increase provider referrals for testing (*P* = 0.121) or patient testing uptake (*P* = 0.159). Of three studies evaluating pre and post multifaceted interventions, two increased TB infection testing (from 0% to 77%, p < .001 and RR 2.28, 1.08–4.80) and one increased provider referrals for TST (RR 24.6, 3.5–174). In another pre-post study, electronic reminders to providers increased reading of TSTs (RR 2.84, 1.53–5.25), but only to 25%. All seven studies were at high risk of bias.

**Conclusions:**

Multifaceted strategies targeting providers may improve targeted TB infection testing in non-U.S.-born populations visiting U.S. healthcare facilities; uncertainties exist due to low-quality evidence. Additional high-quality studies on this topic are needed.

## Background

*Mycobacterium tuberculosis* disease (TB) remains a serious global health problem. In 2017, there were an estimated 10.0 million new cases globally and 1.6 million TB-related deaths, of which 1.3 million were among persons without HIV and 300,000 were among persons living with HIV.[1] In the United States (U.S.), TB incidence declined significantly over the past two decades, from 6.6 per 100,000 persons in 1998 to 2.8 in 2017.[2] Despite this progress, TB remains a public health concern in the United States, where 70% of reported TB cases in 2017 were among non-U.S.-born persons.[2] Untreated latent tuberculosis infection (LTBI) confers an estimated risk of reactivation to TB disease of .098 per 100 person years among non-U.S.-born persons.[3] According to the 2011–2012 U.S. National Health and Nutrition Examination Survey (NHANES), a nationally representative survey, reactivity to the tuberculin skin test (TST) for TB infection, an indicator of LTBI prevalence, was estimated at 5% across all populations and 21% in non-U.S.-born persons.[4] A recent study looking at genotyped TB cases in the U.S. from 2011 to 2014 estimated that 14% of TB cases resulted from recent transmission.[5] Among non-U.S.-born persons only 8% of cases were estimated to result from recent transmission (compared to 27% among U.S. born persons), suggesting that, if one assumes that cases not attributed to recent transmission are due to reactivation of LTBI, 93% of genotyped cases among non-U.S born persons were due to reactivation of LTBI (compared to 73% among U.S. born persons).[5] Thus, non-U.S.-born persons have a higher prevalence of LTBI reactivation relative to US born persons.[5] Fortunately, LTBI reactivation to TB can be prevented through LTBI treatment.

Medical evaluation and treatment for TB disease before entering the U.S. is required for persons seeking permanent U.S. residence (i.e., immigrants and refugees), but not for other visa categories (e.g., students, skilled workers and tourists).[6] The medical evaluation includes review of medical history; physical examination; interferon gamma release assay (IGRA) when indicated; chest radiograph when indicated; and sputum smears and culture testing for *Mycobacterium tuberculosis*. As of 2018, TB infection testing using IGRA is also required for visa applicants aged 2–14 years (but not for other ages) from countries with annual TB incidence of ≥20 per 100,000 population. Prior to that, from 2009–2017, TST or IGRA was acceptable, and currently TST could be used if IGRA is not licensed in the country.[6] LTBI is diagnosed if an asymptomatic patient has a positive TST or IGRA; a chest radiograph not suggestive of TB; and no known HIV infection. Self-reported HIV-positive patients should undergo additional testing to rule out TB disease.

CDC and the U.S. Preventive Services Task Force recommend that U.S. healthcare providers offer targeted testing and treatment (TTT) for LTBI to non-U.S.-born persons and other populations at increased risk of LTBI.[7–9] Yet many non-U.S.-born persons are still unaware of their LTBI since providers fail to test them.[10–12] Our recently published systematic review [13] examined community-based strategies for TTT of non-U.S.-born persons. However, since many non-U.S.-born persons, especially those with legal status, are integrated into mainstream medical care, this study identifies effective strategies to improve TB infection testing and linkage to care in non-U.S.-born populations in healthcare settings.

### Objective

To evaluate the effectiveness of healthcare facility-based strategies to improve targeted TB infection testing and linkage to care among non-U.S.-born populations in the U.S. from published studies.

## Methods

We conducted a systematic review following *Cochrane Handbook[14]* guidance in developing and conducting searches, selecting studies for inclusion, extracting data and assessing risk of bias. We followed reporting guidance in the Preferred Reporting Items for Systematic Reviews and Meta-Analyses (PRISMA).[15] We registered our review protocol in the PROSPERO online registry (registration number CRD42016038476).

Eligible study designs included randomized controlled trials (RCT), quasi-RCTs (RCTs lacking proper randomization), and non-randomized controlled trials with parallel or historical (pre-post) comparator or control conditions. We excluded descriptive studies lacking baseline data; studies of diagnostic test accuracy; and non-U.S. studies. Eligible strategies addressed at least one outcome from the testing portion of the LTBI TTT cascade in non-U.S.-born asymptomatic patients, and were aimed at healthcare providers, non-U.S.-born patients, or both. Eligible provider populations were physicians, nurse practitioners, registered nurses, physician assistants and other clinically trained personnel. Eligible healthcare facilities included primary care outpatient clinics, specialty referral clinics, office-based medical practices, hospitals (including emergency departments) and community-based health centers.

We included studies that explicitly reported that at least some of their participants were born outside of the U.S, regardless of their age of arrival. We excluded studies in “refugee” populations, as these patients are required to undergo pre-departure evaluation for TB before leaving their home countries and are evaluated again once in the U.S.[16] We also excluded studies in which offering and receiving TB infection tests was mandatory (e.g. testing in prisons or required employee testing). Additionally, we excluded studies where TST or IGRA testing was used primarily as part of a diagnostic workup in persons with symptoms consistent with active TB disease. Studies in which TB medical risk factors alone (e.g. diabetes, HIV, rheumatoid arthritis) triggered provider offers of TB infection testing were also excluded because being a non-U.S.-born person is a hard-to-ascertain demographic risk factor in healthcare settings while those with medical conditions can easily be identified and targeted for LTBI screening. Studies where mass testing was offered were excluded as well, unless testing based on non-U.S.-born status was done with results reported separately for non-U.S.-born individuals.

To be included, studies needed a comparator (baseline data for single arm studies or a parallel arm with a control condition) and had to report at least one step in the testing cascade: 1) proportion of eligible non-U.S.-born patients screened or identified for TB infection testing; 2) proportion of providers offering TB infection testing to non-U.S.-born patients; 3) proportion of non-U.S.-born patients receiving TB infection testing; or 4) proportion of non-U.S.-born patients receiving TB infection testing results. The unit of analysis could be the individual patient, individual provider or the healthcare facility. Finally, we included published, “in press,” and unpublished (grey) studies in any language.

### Searches, screening and data collection

We developed a comprehensive search strategy and searched the Cochrane Central Register of Controlled Trials, EMBASE, PubMed and Web of Science. Our search strategy included Medical Subject Heading (MeSH) terms and keywords relevant to TB. The search period ranged from the earliest records to the search date (28 March 2016). We ran updated searches on December 17, 2018, to capture new research published while this manuscript was in development. See “S1 Appendix” for our original and updated database search strategies.

We also searched all available abstracts from the American Public Health Association (APHA) (2000–2018) and the International Union Against TB and Lung Disease North America Region (2014–2018) conferences. Selected “grey literature” (e.g. doctoral dissertations indexed in ProQuest, CINAHL, and WorldCat databases, all issues of TB Notes,[17] government reports and other potentially relevant research) was also searched. As with our main database search, we reviewed grey literature in 2016 and then reviewed newly published material in 2018. We also searched for ongoing studies indexed in the clinical trials registry at the U.S. National Institutes of Health and emailed TB experts to see if they knew of ongoing or completed research that we might have missed.[18] We also searched the bibliographies of included studies as well as the articles that cited them for additional eligible studies.

We imported search results into bibliographic citation management software[19] and excluded duplicate references.[19] Two of three reviewers (AM, HH or JB) independently examined titles and abstracts to identify potentially eligible reports and then independently assessed each full text article and applied inclusion criteria to determine study eligibility. We resolved any differences of opinion through discussion.

Two authors (AM, JB) independently extracted data from each study, entered these data into standardized, piloted data collection forms and compared extracted data. Data collection forms captured details of study populations, intervention characteristics, study design, results for specific outcomes of interest and details necessary to assess risk of bias. We contacted corresponding authors of included studies for additional data when needed.

### Critical appraisal

We used the Cochrane Collaboration tool for assessing risk of bias in included studies.[14] This instrument assesses bias risk in terms of sequence generation, allocation concealment, blinding, incomplete outcome data, selective outcome reporting and other potential biases. With non-randomized studies, we additionally considered socio-demographic comparability of study groups at baseline, potential for measurement bias, adequacy of measures to control for confounding and adequacy of time for intervention follow-up.

### Data analysis and synthesis

We used Stata to calculate cascade step proportions, risk ratios (RR) and their 95% confidence intervals (CI) to measure effectiveness, as well as p-values for differences with comparator arms. [20] We did not conduct meta-analysis to pool data since identified data were too heterogenous and not comparable.

### Evidence quality

We used the Grading of Recommendations, Assessment, Development and Evaluations (GRADE) methodology to assess the quality of evidence for each pre-specified outcome across the literature.[21] In GRADE, “quality of evidence” is defined as “the extent of our confidence that the estimates of effect are correct."[14] The quality rating across studies has four levels: high, moderate, low, or very low. Data from RCTs are initially considered to be of high quality but can be downgraded for five reasons: 1) risk of bias; 2) indirectness of evidence; 3) unexplained heterogeneity or inconsistency of results; 4) imprecision of results; or 5) high probability of publication bias. Data from non-randomized controlled trials are considered to be of low quality but can be upgraded for three reasons: 1) large magnitude of effect; 2) improved outcomes despite plausible confounders that would be expected to worsen outcomes; or 3) the presence of a dose-response gradient.

## Results

**Fig 1:**
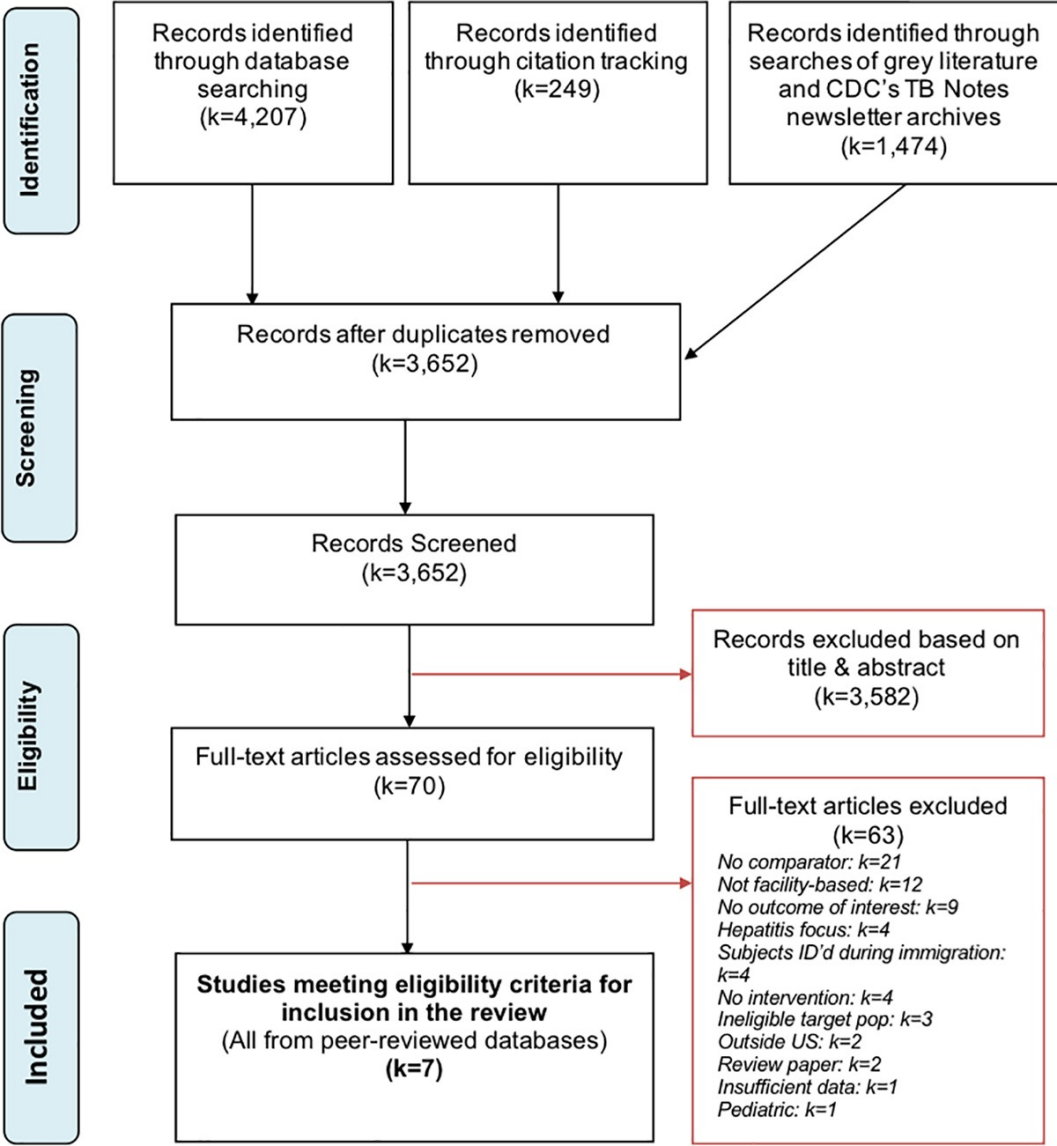
Identification and screening of citations: Systematic review of healthcare facility-based strategies to improve targeted testing for latent tuberculosis infection in non-U.S.-born populations in the United States.

The original and updated searches yielded a total of 3,652 unique records. Based on eligibility criteria, we excluded 3,582 records through examining titles, abstracts and indexing terms. We obtained 70 articles for full-text review, and ultimately excluded 63 of those articles (justifications for exclusion indexed in “S2 Appendix”). We included seven studies concerned with targeted TB testing in non-U.S.-born populations in the U.S., including two exclusively non-U.S.-born studies [22, 23] and five studies [24–28] with some U.S. born participants. See Fig 1 for a PRISMA flow-chart of our screening process and “S1 Prisma Checklist” for the Prisma Checklist.

### Descriptive summary and results from included studies

#### Studies targeting healthcare providers and clinic personnel

**Fig 2:**
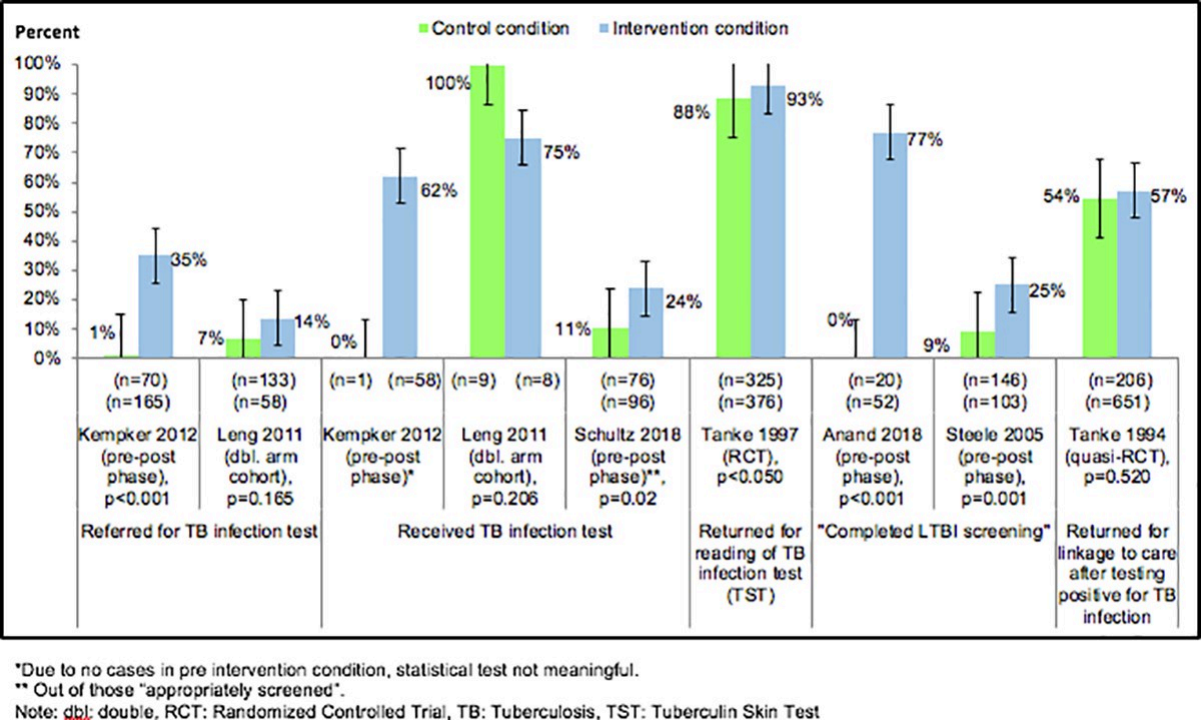
Effect of healthcare facility-based strategies on outcome of targeted latent tuberculosis infection testing cascade in non-U.S.-born populations: referral for testing, receipt of test, tests results read and linkage to care. LEGEND: Green = control condition, Blue = intervention condition.

Steele and colleagues (2005) reported a pre-post study they conducted from 2002–2003 in two community health clinics in Denver, Colorado.[24] Investigators aimed to assess provider compliance to CDC Targeted Tuberculin Testing and Treatment of Latent Tuberculosis Infection guidelines through implementation of a computerized clinical decision support system (CDSS).[8] The CDSS was designed to trigger printed alerts for placement in patients’ records when their personal information indicated that they were at high risk of LTBI according to the CDC guidelines. There was also a web-based data entry component to walk the healthcare providers through the TB infection testing process once an alert was triggered. Additional intervention components are described in Table 1. Investigators reviewed a random sample of 249 non-U.S.-born patient charts (97% born in Mexico) to assess provider compliance with the CDC guidelines measured by the proportion of eligible patients returning for TST interpretation. Of the 249 patient charts examined, investigators assessed provider compliance with 146 (59%) patients in the pre-CDSS period, and 103 (41%) patients after (see Fig 2). Following CDSS implementation, provider compliance improved from 9% (13/146) to 25% (26/103), (RR 2.84, CI 1.53 to 5.25; p<0.001), leaving 75% as non-compliant.

**Table 1:** Characteristics of healthcare facility-based targeted TB testing studies non-U.S.-born populations in the US.

Author/ Year/Publication type	Design	Setting	Intervention & comparator	Outcome and characteristics
**Studies targeting providers and other clinic personnel**				
**Steele 2005** Peer Reviewed Article	Pre-post	Colorado, 2002–2003	**Electronic health record prompts vs. no prompts** In the intervention phase, when providers encountered patients <40 years of age, who were born in a high-risk TB country, they received on-screen prompts for TB screening in the patients’ electronic health records (EHR), followed by guided web-based documentation. Clinic personnel also received computer-generated paper alerts.	**Outcome analyzed:** “completed LTBI screening” which entailed the use of a computerized clinic decision support system that alerted providers when a patient was at high risk of LTBI, the provider offering a TB infection test and then placing the test in consenting patients. **Analysis sample**: n = 8463. **Demographics:** Investigators do not report the number of providers evaluated. No provider demographic details reported. All patients were non-U.S.-born; 97% were born in Mexico.
**Kempker 2012** Peer Reviewed Article	Pre-post	Georgia, 2009–2010	**Multifaceted organizational improvement vs. previous organization and practice standard** The intervention involved a nurse administered questionnaire placed in patient files for physicians to review and, if appropriate offer TB infection testing. Other components: training of clinic personnel and coordination with relevant county health departments.	**Outcome analyzed:** referred for TB infection test and received TB infection test. **Analysis sample:** asymptomatic patients, n = 165. **Demographics:** The entire sample was non-U.S.- born: 58% were from Mexico; 7% were from Colombia; and 35% came from other countries. Mean age: 46 ± 14 yrs (range 20–85 yrs). 70% female. Mean time in U.S.: 11 ± 6.8 yrs. Mean household size: 4 ± 2.0.
**Schultz 2018** Conference Abstract	Pre-post	Colorado, 2016–2017	**Multifaceted education, reminder and posted screening guidelines vs. no education session, reminder or posted guidelines.** In the intervention phase, medicine-pediatrics (Med-Ped) residents and staff at Denver Hospital attended an education session and received an email reminder to screen for LTBI. Additionally, a flowchart with screening guidelines was visibly posted in the resident/staff area of the clinic.	**Outcome analyzed:** proportion of individuals who were administered an TB infection test among those who were identified as being at high risk of LTBI and appropriately screened. **Analysis sample:** clinic patients meeting LTBI screening guidelines, n = 172. **Demographics:** pre-intervention phase: n = 76, mean age: 44 (range 2–77 yrs). 62% Female. post intervention phase: n = 96, mean age: 46 (range 2–93 yrs). Female: 59%.
**Anand 2018** Peer Reviewed Article	Pre-post	Florida, 2015–2017	**Multifaceted quality improvement project vs. previous standard practice.** The intervention included an educational training for providers and staff in a free student-run clinic as well as the introduction of an LTBI screening tool (questionnaire) adapted from CDC LTBI screening guidelines	**Outcome analyzed:** “screened for LTBI” which entailed using a screening tool (questionnaire) adapted from CDC guidelines and, when deemed appropriate offering a TB infection test and then placing the test in consenting patients. **Analysis sample:** clinic patients, n = 72 (20 before and 52 after intervention). **Demographics** (only reported for post/intervention arm): 5% non-U.S.-born; 30% “emigrated from endemic region”.
**Studies targeting patients**				
**Tanke 1994** Peer Reviewed Article	Quasi-RCT	California, 1992	**Telephone reminder vs. no reminder.** In the intervention arm, patients who had a positive TST were sent one of four types of reminders the evening before they were due to attend their next appointment for a chest x-ray and LTBI treatment evaluation. The control arm received no reminders. Reminders could be ‘basic reminders’ or have additional enhancement (e.g. include an authority endorsement). All patients were given a copy of the clinic’s schedule and verbally told which day to return. All reminders “were recorded by a female speaker, in participants' home language, b) identified individuals by name, and c) gave the time of appointment, clinic address and phone number of clinic, d) reminded participants to bring along the record given at time of administration of test, and e) indicated that the test would have to be repeated if the reading was not taken the following day.”	**Outcome analyzed:** returned for TB infection test (TST) reading. **Analysis sample:** Asymptomatic patients (n = 858). **Demographics of entire study sample:** (n = 2008) “home language” of participants: 39% Spanish; 28% Vietnamese; 6% Tagalog; 14% English; and 14% spoke one of "two other languages". Median age 19 (range 0–81 yrs). 46% female.
**Tanke 1997** Peer Reviewed Article	RCT	California, year not reported	**Telephone reminder vs. no reminder.** Patients in the intervention arm received a telephone reminder to return to the clinic to have their TST read and a warning that if they did not return in the designated time frame, they would need to have a new test placed. Reminders were of one message type, not described in the report but likely to be of the “basic” message type.	**Outcome analyzed:** returned for TB infection test (TST) reading. **Analytic Sample:** Asymptomatic adults and children (n = 701). **Demographics:** “Home language”: Spanish 29%; Vietnamese 3%; English 68%. Age ≤13: 55%. Age ≥20: 27%. 55% female.
**Leng 2011** Peer Reviewed Article	Retrospective cohort	New York, 2003–2005	**Language-concordant patient encounters vs. language-discordant patient encounters.** Patients in the intervention arm were offered language-concordant patient encounters (in which providers and patients spoke the same language, and jointly decided not to use an interpreter) while those in the control arm received language-discordant patient encounters (in which providers and patients did not speak the same language and used the services of an interpreter.	**Outcome analyzed:** referred for TB infection test and received TB infection test **Analysis sample:** n = 191. **Demographics**: All participants were non-U.S.-born patients arriving to the U.S. in past five years. Primary language: 68% Spanish and 29% Mandarin or Cantonese. None spoke English as primary language. Of language concordant encounters, 71% were Spanish concordant, 16% were Mandarin or Cantonese concordant and 14% were English concordant.

From 2009–2010, Kempker and colleagues (2012)[22] implemented a multifaceted performance improvement intervention in Atlanta, Georgia, targeting healthcare providers and other clinic personnel.[22] The goal of the intervention was to improve identification of those at increased risk of TB infection (active TB disease or latent TB infection) and subsequent referral for TB infection testing. The evaluation compared pre- to post-intervention proportions. Following CDC guidelines, investigators developed a questionnaire to identify non-U.S.-born patients at increased risk of TB infection, which was administered by a nursing assistant during patient intake and placed in the patient’s medical records for review by the healthcare provider during the appointment. When appropriate, providers referred patients for a TST based on the questionnaire. The study sample was entirely non-U.S.-born; fifty five percent of patients in the post intervention condition were from Mexico; country of origin of patients before the intervention was not reported. Of 71 non-U.S.-born patient charts reviewed before the intervention, one patient was excluded from analysis because he already received a diagnostic TB test and one patient (1%) was referred for TB infection testing by the provider but did not receive a TST. In the seven months following the start of the intervention, out of the 165 non-U.S.-born patients who met TB risk criteria, 58 (35%) were referred by their provider to receive a TST. Thirty-six (62%) of those referred followed through and received a TST, two of whom were ultimately diagnosed with TB. The intervention improved referral for TST (RR 24.6, CI 3.5 to 174.1; p = 0.001). Statistical assessment of change in receiving TST was not meaningful due to lack of cases at baseline.

Schultz and colleagues (2018) conducted a pre-post study at the Denver Hospital Internal Medicine and Pediatrics (Med-Peds) resident clinic from October 2016 through June 2017.[25] The clinic is located in Southwest Denver and primarily serves low-income Hispanic patients, including adults and children. The intervention was a multifaceted quality improvement project targeting clinic providers and staff, consisting of one education session on LTBI; an email reminder to clinic providers about screening for LTBI; and an LTBI screening flowchart posted in the resident area of the clinic. The purpose of the intervention was to improve (i) identification of non-U.S.-born persons at risk of LTBI and (ii) TB infection testing of those at risk of TB using QuantiFERON®-TB (QFT). Limited demographics on study participants were reported. The analytic sample (n = 172) included participants who met CDC guidelines for being at risk of LTBI.[29] Reported results were not stratified by age group. The mean age of patients in the pre-intervention group (n = 76) was 44 years (range 2–77) and 62% were female. In the post-intervention group (n = 96), the mean age of patients was 46 years and 59% were female. Outcomes reported included the proportion of individuals identified as being at risk of LTBI who received a QFT, and the proportion who tested positive and received a chest x-ray. Criteria for identifying who was “at risk of LTBI” were not described in the paper. Only one outcome (the proportion who received a QFT) was reported for both pre- and post-intervention conditions. During the pre-intervention phase (October-November 2016), 11% (8/76) of patients who had at least one LTBI risk factor were screened for LTBI via QFT administration. During the post-intervention phase (May-June 2017), 24% (23/96) of patients received a QFT. Patients in the intervention arm were more than twice as likely to be screened, a statistically significant difference (RR = 2.28, CI 1.08 to 4.80; p = 0.023).

Anand and colleagues (2018) conducted a pre-post study from December 2015 to February 2017 at The Keeping Neighbors In Good Health Through Service (KNIGHTS), which provides free care to underserved populations in Orange County, Florida.[26] The pre-post study was a multifaceted quality improvement project aimed at improving provider and staff compliance to CDC LTBI screening guidelines.[29] The intervention consisted of educational training sessions for providers and clinic staff as well as the development of an LTBI screening tool (questionnaire) for clinic use, which was adapted from CDC LTBI screening guidelines. The questionnaire was administered by providers three months after the training, and responses were added to patient medical records to identify patients recommended to be offered a TB infection test. The paper also reported treatment initiation and retention among those with a positive test. However, the only outcome that was reported pre- and post-intervention was the proportion of clinic patients who were screened for LTBI via the questionnaire and TB infection test. No demographics (n = 72) were reported, other than the proportion of the 52 patients post-intervention with specific LTBI risk factors. In this group, five percent of participants who were screened were non-U.S.-born. Among those testing positive, 30% came from a TB endemic region (as determined from screening tool list, which included Latin America, Caribbean, Africa, Asia, Eastern Europe, Russia and “other”). Since the paper did not describe the demographics of participates further, it is unclear if children were eligible for participation in the study. During the pre-intervention phase, none of the twenty patients were screened for LTBI. Fourteen months after the intervention, three quarters (77%) of 52 patients were screened for LTBI. Due to the small sample size (and no patients screened pre-intervention), a risk ratio for intervention effect was not calculated (p<0.001).

### Studies targeting healthcare patients

In a study conducted in Santa Clara, California, Tanke and colleagues (1994) used a quasi-RCT design to test an automated telephone reminder system for improving patient appointment attendance for chest radiography after receipt of a positive TB infection test.[28] The proportion of non-U.S.-born patients in the overall sample (N = 2008) was not reported, but the primary language spoken by 86% of patients was not English. While the overall sample included both adults and children, the proportion of children was not reported; the median age of participants was 19 years. The study was carried out at three different types of clinics representing different phases in the TB test and treat cascade. We limited our analysis to 857 patients at the “reactor clinic”; these patients were being referred to the “Tuberculosis clinic” where they were to receive chest radiography and evaluation for LTBI treatment after receiving a positive TST result. The demographics of patients referred to the TB clinic for radiography were not reported; demographics for the full sample are reported in Table 1. Over a six-month period, depending on the day of the week, patients in the intervention arm received one of four types of reminders: basic reminder; “authority endorsed” reminder (which stated at the beginning that the message was from a public health nurse at the health department); reminder plus statement of importance (highlighting that appointment attendance was important because it could prevent the patient and their family from becoming seriously ill); and authority endorsed reminder with statement of importance. The reminders were delivered the evening before their scheduled chest x-ray; the control arm received no reminders. While 57% (371/651) of patients receiving any reminder attended the TB clinic for their chest radiography and LTBI treatment evaluation, so did 54% (111/206) of those receiving no message (RR 1.05, CI 0.91 to 1.21, p = 0.520).

In another study, also in Santa Clara County, Tanke and colleagues (1997) conducted an RCT using telephone prompts at two of the largest clinics in the county’s immunization program.[27] Over a seven-week period, all patients who attended and received a TST were given an index card, asked to provide their contact info, and told that they might receive a reminder for a follow-up visit to interpret the TST. Investigators randomly assigned patients to two arms. Patients or guardians in the intervention arm received phone reminder messages the evening before they were due to have the TST interpreted, and those in the control arm did not receive phone messages. This study included adults and children and didn’t stratify results by age group. Fifty five percent of the sample was 13 years of age or less. The manuscript did not report number of patients in each arm or the proportion of the sample that was non-U.S.-born, but 32% of patients were non-English speakers. We identified denominators of the respective arms in a Cochrane review.[30] The study showed that 93% (349/376) returned for TST interpretation in the intervention versus 88% (287/325) in the control arm (RR 1.05, 95% CI 1.00 to 1.10, p = 0.044).

Leng and colleagues (2011) reported findings from a 2003–2005 study testing the use of language interpretation services in a large New York City hospital. In a retrospective cohort nested in an RCT, investigators offered targeted TB testing to persons recently arrived to the United States (n = 191) from settings with high TB endemicity.[31] Patients were offered provider encounters that were either language-concordant (provider and patient spoke the same language and opted out of using an interpreter) (n = 58; intervention) or language-discordant (provider and patient spoke different languages, sometimes with the use of an interpreter) (n = 133; control). The majority (71%) of the language concordant visits were conducted in Spanish; the rest were Mandarin or Cantonese concordant (15%) or English concordant (14%). Patients in both arms did not differ significantly in year of U.S arrival or age. In the concordant arm, 8/58 (14%) were referred for TB infection testing, of whom six (75%) received a TST. In the discordant arm, 9/133 (7%) of patients were referred for TB infection testing, all of whom received a TST. The effect sizes were not statistically significant: referrals for TB infection testing (RR = 2.0, CI 0.83 to 5.02, p = 0.121) and receipt of TST (RR 0.8, CI 0.50 to 1.12, p = 0.159). It is unclear how provider fluency was determined, and it is possible that some providers may have had “false fluency” (i.e., a provider believed to be fluent in a language may have limited fluency in that language, which could hinder the effectiveness of communication in a patient-provider concordant pair); this is one possible explanation for the lower rates of return for testing in the intervention arm.

### Risk of bias in included studies

**Fig 3:**
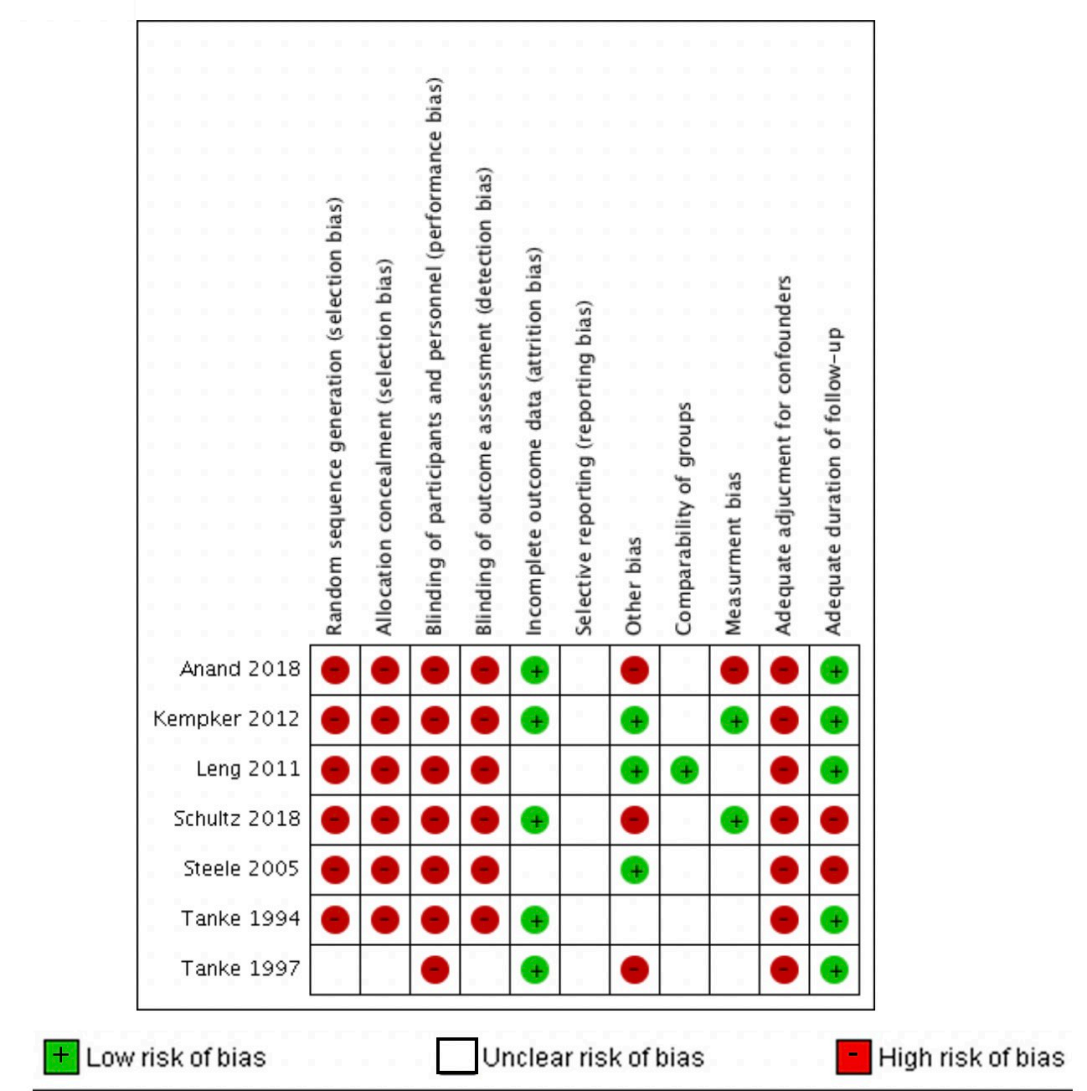
Systematic review of healthcare facility-based strategies to improve targeted testing for latent tuberculosis infection in non-U.S.-born populations in the United States. LEGEND: Green = low risk of bias, White = risk of bias unclear, Red = high risk of bias.

All seven studies were at high risk of bias (Fig 3). In the sole RCT (Tanke 1997), methods for randomizing participants to group and allocation concealment were not described and blinding of participants and outcome assessors was either not done or not described.[27] As no study protocol was available in the Tanke 1997 study, we were unable to ascertain whether or not outcomes were reported selectively. Finally, since the authors are financially invested in a company selling equipment for telephone reminder messages, we considered this study being at high risk of “potential” bias due to conflict of interest, although this does not necessarily mean the study’s findings were affected by this bias.[27] Rates of return for test interpretation were very high in both arms of the RCT; the inclusion of children in the sample may have introduced a potential source of bias; as parents are often more responsive to their children’s medical needs than their own. However, the inclusion of children cannot entirely explain the high rates of return; the original paper reported that the most adherent group was the oldest age cohort (those over the age of 29 years). Tanke 1994, also included children in the sample but didn’t find any significant differences in message effectiveness by age group.[28]

The other six studies were non-randomized.[22, 24–26, 28, 31] Among five,[22, 24–26, 31] none blinded participants, personnel or outcome assessors to the intervention status. This information was unclear in Tanke 1994.[28] With study protocols unavailable, the risk of outcome reporting bias in all studies was also unclear. Three studies[22, 26, 28] used clinic records or administrative data. In two studies,[24, 31] groups were socio-demographically comparable from baseline to intervention, but comparability of groups was unclear because of lack of reported data for both phases in the four others.[22, 25, 26, 28]

With regard to risk of measurement bias, it was low in two studies,[22, 25, 26] unclear in three others,[24, 28, 31] and high in one study.[26] Steele and colleagues assessed outcomes by reviewing random samples of patient charts, but they did not describe their sampling methods. As mentioned in the results section, in Leng 2011, some providers deemed language-concordant may have had “false fluency” in their languages but this was unclear. Referral proportions in both groups were very low, and investigators speculated that among other possibilities, participants may have refused referral. In Tanke 1994, investigators did not report if or how they verified that messages were actually delivered. The study by Anand et al. was ranked as high for risk of measurement bias because it is unclear what tools or protocols (if any) were being used to document patients who were screened for LTBI prior to the intervention.

Four studies (Kemper et al., Anand et al., Tanke 1994, and Leng et al.)[22, 26, 28, 31] were considered at low risk of bias for follow-up duration. Tanke 1994 and Leng et al.[28, 31] both targeted patients for short-term behavior change or after behavior modification (such as attending an appointment for a test or receiving language concordant care at a single visit). The participants were followed long enough to capture the outcomes of interest and were therefore viewed as having an adequate follow-up duration. Kempker et al., Anand et al., Shultz et al. and Steele et al. all targeted providers and clinic personnel.[22, 24–26] Kempker et al. followed providers for five months after intervention implementation and Anand et al. followed them for fourteen months [22, 26]; both of these studies were considered to be at low risk for follow-up bias because these follow-up durations were considered sufficient (> 3 months) to determine if the intervention had changed provider and clinic personnel behaviors around screening. Shultz et al. and Steele et al. were both considered to be at high risk because the follow-up period was < 3 months, which was considered insufficient to measure true behavior change retention of provider screening practices.[24, 25] Finally, all six non-randomized studies (all included studies except Tanke 1997) were at high risk of bias for not controlling for potential confounders. See Fig 3 for a summary assessment of bias risk in each included study.

#### Quality of the evidence

The evidence quality for all interventions reviewed here is very low. Thus, we can have little confidence in the stability or accuracy of any effect estimate. All studies were at high risk of bias, including the only RCT. We down-graded the evidence quality in Tanke (1997) to very low (by three levels), for lack of blinding of patients, personnel and outcome assessors and for high risk of bias due to conflict of interest.[27] We also judged the other six non-randomized studies as providing low-quality evidence, because they were non-randomized, none of them reported using statistical methods to adjust for confounders, and one had imprecision due to the small numbers of participants.[26]

## Discussion

Despite systematically reviewing the scientific literature to find studies to improve TB infection screening of non-U.S.-born populations in healthcare settings in the U.S., we identified only seven studies, all of poor methodologic quality. Three of these studies [27, 28, 31] targeted patients to improve targeted TB infection testing and linkage to care while four targeted providers and clinic personnel.[22, 24–26] Two of the studies targeting patients used telephone reminders to remind patients to return for their next appointment. Tanke et al. (1997)[27] saw statistically significant differences in rates of return for TST interpretation but in their earlier study (1994) Tanke et al. saw no significant difference in rates of chest x-ray appointment attendance between participants in the intervention arm and those in the control arm.[28] The four interventions[22, 24–26] targeting providers all had statistically significant improvements in the intervention arm for at least one of the outcomes of interest reported.

Of the interventions targeting providers, three implemented a multifaceted “quality improvement” intervention, each comprised of education and training for clinic staff and providers; the development of LTBI risk screening materials (questionnaires and visibly posted screening guidelines) for the clinic; and reminders to provide a TB infection test for those who are deemed “at risk”. Kempker et al.’s intervention saw the largest effect size (RR 24.6 CI 3.5 to 174.1; p = 0.001 for referral for TB infection testing), but given the wide confidence interval, caution is needed when interpreting these results.[22] Anand et al., who implemented a similar intervention with an education component to improve provider administered LTBI risk screening (through a questionnaire and when warranted, TB infection testing), saw a statistically significant increase in rates of provider risk screening (p<0.001), but a reliable effect size could not be calculated due to the small sample size in the pre-intervention arm.[26] Similarly, Schultz et al. saw statistically significant improvements in receipt of IGRA TB infection test among those identified as being “at risk” of LTBI (p<0.001).[25] The final study aimed at providers (Leng 2011) used a clinical decision support system to flag patient’s medical files if they should be tested for TB infection.[31] This study assessed differences in the rate of TB infection testing referral and testing uptake among those in the intervention and control arm. While there was a statically significant improvement in rates of referral in the post-intervention arm, the improvement in TB infection testing rates was not significant.

Although healthcare facilities play an important role in dealing with the LTBI epidemic among non-U.S.-born persons, there is a paucity of studies that rigorously assessed the effect of healthcare facility-based interventions to improve outcomes along the LTBI test and treat cascade among this population in the U.S.. Comparative studies that utilize techniques such as randomized allocation of intervention and blinding are needed to produce reliable evidence for these interventions. While other systematic reviews mentioned below have looked at the value of targeted TB infection testing and treatment among populations at high risk of LTBI, to our knowledge this is the only systematic review that required a comparator arm and focused on the testing portion of the TTT cascade in healthcare facilities in the U.S. Importantly, LTBI diagnostics and treatment regimens have changed over the time period of this review and these changes could impact willingness to be tested and referred to treatment. In 2001, QuantiFERON®, an interferon gamma release assay (IGRA) was approved by the FDA for TB infection testing, eliminating the need to return to have test results read 48–72 hours after having a TST placed. However, TST remains common and all but one of the studies included in this review relied on it for testing.

### Evidence from other diseases

The strategies used to improve targeted TB testing in reviewed studies are not unique to TB and have been successfully used to improve screening and testing uptake for other chronic infections such as hepatitis B virus (HBV) and hepatitis C virus (HCV). For instance, an RCT among providers in 15 primary care clinics in California found that the study arm that received “alerts” to screen for HBV when a patient’s surname was of Chinese or Vietnamese origin (vs no alert) improved proportion of patients who were offered testing (36/88 vs 1/87; p<0.001) as well as testing completion (30/36 vs 0/1; p<0.001).[32] HepCAT, a comparable multifaceted intervention to improve targeted HCV testing using “risk-based” sticker alerts in patient charts, in conjunction with patient, provider and staff knowledge transfer and HCV awareness posters, saw an improvement of testing among patients with ≥1 risk factor from 5% to 14% after 15 weeks of the intervention (n = 7846).[33, 34]

In the absence of sufficient TB infection screening specific literature, high quality systematic reviews of strategies aiming to improve health outcomes through organizational changes or enhancing provider practice or behavior may provide insights to design and test interventions to improve targeted TB infection testing. A Cochrane systematic review[35] of continuing education found that it improved provider compliance with desired practice behavior (risk difference [RD] 6.0, interquartile range [IQR] +2% to +15%). Electronic prompts and reminders in health records led to a 5% (IQR +3% to +11%) median absolute improvement of care in another Cochrane review.[36] A Cochrane review[37] of audit and feedback strategies found a weighted median adjusted RD of 4% (IQR +1% to +16%) in provider compliance with desired practice.

With regard to improving patient uptake of screening or tests, a few systematic reviews have examined existing strategies.^37^ A Cochrane review^38^ of personalized risk communication with providers improved patients’ informed decision-making about screening (odds ratio [OR] 3.65, 95% CI 2.13 to 6.23). Another Cochrane review^39^ of “decision aids” improved patient knowledge about screening (mean difference [MD] 13.34/100; 95% CI 11.17 to 15.51).

### Limitations of this review

Our findings should be interpreted with caution for several reasons. First, we found few eligible studies. Despite exhaustive searches and rigorous review methods, we could have missed unpublished research conducted in non-U.S.-born populations by state or local health departments. The studies we did find were all at high risk of bias. The generalizability of our findings may be limited due to small sample sizes, lack of demographic data and the heterogeneity of study settings.

Most of the studies we found were pre-post and analyzed administrative data. We believe that when a change is observed from baseline to after intervention, it is likely real. However, we are also aware that such administrative evaluations may get published preferentially when they suggest an effect, since authors are more eager to share what worked and journals are more willing to publish it. Thus, we think there is a large potential for publication bias. Additionally, sample sizes for the pre-intervention arm for some of the studies included were very small, prohibiting our ability to calculate meaningful effect sizes (e.g. Anand).[26] In addition, we expect that changes observed in pre-post data might not be fully a result of the intervention. The change may be partially the result of a contemporaneous event (that may have also instigated the intervention, or just happened to coincide), or due to changes in characteristics of providers or patients. Finally, four of the studies in our review were rather old (data collection concluded prior to 2010), and all but one included study [25] reporting TB infection testing outcomes used TST, which limits the applicability of our findings to testing programs that are increasingly using IGRA.

## Authors’ conclusions

While the quality of evidence from the included studies was very low and data were sparse, our findings suggest that multifaceted strategies combining media, patient and/or provider education and staff training on screening for risk of LTBI, as well as those that utilize automated patient appointment reminders, show promise. All of the studies targeting providers saw significant improvements in the intervention arm for at least one reported step in the testing cascade while only one of the studies targeting patients saw significant improvement, suggesting interventions targeting provider behavior change may have a greater impact. Our certainty about the stability or accuracy of any effect estimate from the studies included in this review is very limited. All of the studies captured in this review targeted either patients or providers to improve screening for LTBI risk factors or TB infection testing among those identified as being “at risk” of LTBI. Additional high-quality studies are urgently needed to examine this important public health issue more closely. Future studies should explore combining these two approaches (patient-centered and provider-centered approaches) for a more robust intervention. Additional rigorous research (in the form of randomized controlled trials) is needed to ascertain the most effective strategies to improve targeted TB infection testing and reduce the burden of LTBI in the U.S., and comparative evaluations are needed to substantiate these findings. At minimum, future studies that do not use a randomized study design should ensure sufficient demographic data is collected for the control/pre-intervention arm to allow for meaningful comparison of intervention effect across groups.

### Public health implications

There is a need for rigorous research including control or comparison populations of innovative strategies to improve TB infection testing in non-U.S.-born populations in healthcare settings. These strategies can contribute to TB elimination efforts.

## Supporting information

S1 Prisma Checklist (DOC)

S1 Appendix (DOCX)

S2 Appendix (DOCX)

S3 Appendix (XLSX)
