# Supplementary material for: Healthcare facility-based strategies to improve tuberculosis testing and linkage to care in non-U.S.-born population in the United States: A systematic review
Source: PLoS One. 2019 Sep 30;14(9):e0223077. doi: 10.1371/journal.pone.0223077 (PMC6768470; doi:10.1371/journal.pone.0223077)
Supplement: S1 Appendix — (DOCX) [file pone.0223077.s002.docx]

# **S1 Appendix: Database search strategies**

**A preliminary note about our search strategies:** In addition to terms designed to capture reports of tuberculosis (TB) testing studies, our search strategies included terms relevant to hepatitis B virus (HBV) and hepatitis C virus (HCV) infections. This note explains the inclusion of these terms. We specified in our protocol that we planned to include only studies concerned with targeted TB testing in non-U.S.-born populations. However, preliminary “scoping” searches of the scientific literature suggested a paucity of evidence for strategies to improve targeted TB testing outcomes in this population in healthcare settings in the U.S. Therefore, we took the decision *a priori* that if we identified fewer than four TB testing studies that directly met inclusion criteria, we would consider including indirect evidence from studies focused on improving testing rates in non-U.S.-born patients for other “hard-to-treat disease with high burden affecting this population.” We specified this in our protocol. In the context of our research question, “hard-to-treat, high non-U.S.-born burden” diseases would have these characteristics: 1) more prevalent in non-U.S.-born persons coming from countries with a high burden of that disease, compared to the US burden; 2) have a ≥6 month asymptomatic period, during which screening may be performed; 3) require a relatively long (≥2 months) and uninterrupted treatment period; and 4) may lead to worsened health if left undiagnosed and untreated. We found that hepatitis B virus (HBV) and, to a smaller degree, hepatitis C virus (HCV) infections met these criteria. If targeted TB testing studies had proven to be sparse and HBV or HCV testing studies otherwise met inclusion criteria, we would have included the HBV and HCV studies in this review. However, we identified five TB testing studies, so we included no HBV or HCV studies and when we updated our searches in 2018, we omitted search terms related to HBV and HCV. We searched using the term “foreign-born” in our database searches, but for reporting purposes, we use “non-U.S.-born” in the manuscript.

In 2018, we sought to capture research published after the original search. In order to capture the most relevant TB studies efficiently, we modified our original search strategy. The original strategy had defined six topic-specific queries and combined sets of them in three different ways. Our updated strategy combined all six queries in one statement, returning fewer, but more focused results. We reviewed these results to determine if this modification would have captured all of the studies identified in our original search. Where those studies were not captured, we identified the additional keywords and subject headings necessary and appended those to the relevant topical queries. We then limited this new search to the period from 29 March 2016 onward (or, where databases permitted limits by year, from 2016 onward). We report both searches below.

**Original Search Strategy**

**Our database searches were conducted on 29 March 2016. Total records retrieved: 3636. After removing 954 duplicate records, we screened 2682 unique records obtained through database searches.**

| **Search** | **29 March 2016 PubMed query (k=1073)** |
| --- | --- |
| **#10** | **#7 OR #8 OR #9** |
| #9 | #1 AND #3 AND #4 AND #5 |
| #8 | #1 AND #4 AND #5 |
| #7 | (#1 AND #2 AND #3 AND #4 AND #5) **OR** (#3 AND #4 AND #5 AND #6) |
| #6 | Migrant*[tiab] OR immigrant*[tiab] OR foreigners[tiab] OR foreign-born[tiab] OR refugee*[tiab] OR high risk population*[tiab] OR overseas[tiab] OR chinese[tiab] OR mexican[tiab] OR guatemalan[tiab] OR vietnamese[tiab] OR haitian[tiab] OR philippines[tiab] OR china[tiab] OR mexico[tiab] OR guatemala[tiab] OR haiti[tiab] OR vietnam[tiab] OR “viet nam”[tiab] OR Filipino[tiab] OR Emigrants and Immigrants[mh] |
| #5 | Mass screening[mh] OR screening[tiab] OR diagnosis[mh] OR diagnosis[tiab] OR diagnostic[tiab] OR screen*[tiab] OR test*[ti] OR uptake[tiab] OR recruit*[tiab] OR linked[tiab] OR linkage*[tiab] |
| #4 | tuberculosis[mh] OR tuberculosis[tiab] OR tb[tiab] OR latent tuberculosis infection[mh] OR ltbi[tiab] OR hepatitis[mh] OR hepatitis[tiab] OR hbv[tiab] OR hcv[tiab] |
| #3 | “united states”[mh] OR “united states”[tiab] OR alabama[tiab] OR alaska[tiab] OR arizona[tiab] OR arkansas[tiab] OR california[tiab] OR colorado[tiab] OR connecticut[tiab] OR delaware[tiab] OR florida[tiab] OR georgia[tiab] OR hawaii[tiab] OR idaho[tiab] OR illinois[tiab] OR indiana[tiab] OR iowa[tiab] OR kansas[tiab] OR kentucky[tiab] OR louisiana[tiab] OR maine[tiab] OR maryland[tiab] OR massachusetts[tiab] OR michigan[tiab] OR minnesota[tiab] OR mississippi[tiab] OR missouri[tiab] OR montana[tiab] OR nebraska[tiab] OR nevada[tiab] OR “new Hampshire”[tiab] OR “new jersey”[tiab] OR “new mexico”[tiab] OR “new York”[tiab] OR “north Carolina”[tiab] OR “north Dakota”[tiab] OR ohio[tiab] OR oklahoma[tiab] OR oregon[tiab] OR pennsylvania[tiab] OR “rhode island”[tiab] OR “south Carolina”[tiab] OR “south Dakota”[tiab] OR tennessee[tiab] OR texas[tiab] OR utah[tiab] OR vermont[tiab] OR virginia[tiab] OR washington[tiab] OR “west Virginia”[tiab] OR wisconsin[tiab] OR Wyoming[tiab] |
| #2 | practice guideline*[tiab] OR physicians[mh] OR physician*[tiab] OR provider[tiab] OR providers[tiab] OR clinician[tiab] OR clinicians[tiab] OR “physician assistants”[mh] OR physician assistant*[tiab] OR nurse[mh] OR nurse*[tiab] OR nurse practitioners[mh] OR nurse practitioner*[tiab] OR "Academic detailing”[tiab] OR continuing education[tiab] OR “audit and feedback”[tiab] OR reminder*[tiab] OR opinion leader*[tiab] OR incentive*[tiab] OR training[tiab] OR multifaceted[tiab] OR office-based[tiab] OR organizational[tiab]OR Physician's Practice Patterns[mh] OR practice patterns[tiab] OR guideline adherence[mh] OR clinic*[tiab] OR facility OR facilities[tiab] OR [tiab] OR hospital*[tiab] OR office*[tiab] OR practice*[tiab] OR primary care[mh] OR primary care[tiab] |
| #1 | observational[tiab] OR comparative[tiab] OR randomized controlled trial[pt] OR randomized controlled trial[tiab] OR random*[tiab] OR cohort studies[mh] OR cohort*[tiab] OR intervention[tiab] OR evaluation[tiab] OR compared[tiab] OR trial[tiab] |

| **Search** | **29 March 2016 Embase query (k=851)** |
| --- | --- |
| **#11** | **#10 AND limit: Humans** |
| **#10** | **#7 OR #8 OR #9** |
| #9 | #1 AND #3 AND #4 AND #5 |
| #8 | #1 AND #4 AND #5 |
| #7 | (#1 AND #2 AND #3 AND #4 AND #5) **OR** (#3 AND #4 AND #5 AND #6) |
| #6 | 'migrant'/exp OR 'immigrant'/exp OR 'foreigners' OR 'foreign-born' OR 'refugee'/exp OR 'high risk population'/exp OR overseas OR 'chinese'/exp OR 'mexican'/exp OR 'guatemalan'/exp OR 'vietnamese'/exp OR 'haitian'/exp OR 'philippines'/exp OR 'china'/exp OR 'mexico'/exp OR 'guatemala'/exp OR 'haiti'/exp OR 'vietnam'/exp OR 'viet nam'/exp OR 'filipino':ab,ti |
| #5 | 'mass screening'/exp OR 'screening'/exp OR 'diagnosis'/exp OR 'diagnostic' OR 'screen':ti OR ‘test’:ti OR ‘testing’:ti OR uptake OR recruit OR recruitment OR linked OR linkage |
| #4 | 'tuberculosis'/exp/mj OR 'tuberculosis' OR 'tb'/exp/mj OR 'tb' OR ltbi OR 'hepatitis'/exp/mj OR 'hepatitis' OR 'hbv' OR 'hcv' |
| #3 | 'united states'/exp OR 'alabama'/exp OR 'alaska'/exp OR 'arizona'/exp OR 'arkansas'/exp OR 'california'/exp OR 'colorado'/exp OR 'connecticut'/exp OR 'delaware'/exp OR 'florida'/exp OR 'georgia' OR 'hawaii'/exp OR 'idaho'/exp OR 'illinois'/exp OR 'indiana'/exp OR 'iowa'/exp OR 'kansas'/exp OR 'kentucky'/exp OR 'louisiana'/exp OR 'maine'/exp OR 'maryland'/exp OR 'massachusetts'/exp OR 'michigan'/exp OR 'minnesota'/exp OR 'mississippi'/exp OR 'missouri'/exp OR 'montana'/exp OR 'nebraska'/exp OR 'nevada'/exp OR 'new hampshire'/exp OR 'new jersey'/exp OR 'new mexico'/exp OR 'new york'/exp OR 'north carolina'/exp OR 'north dakota'/exp OR 'ohio'/exp OR 'oklahoma'/exp OR 'oregon'/exp OR 'pennsylvania'/exp OR 'rhode island'/exp OR 'south carolina'/exp OR 'south dakota'/exp OR 'tennessee'/exp OR 'texas'/exp OR 'utah'/exp OR 'vermont'/exp OR 'virginia'/exp OR 'washington'/exp OR 'west virginia'/exp OR 'wisconsin'/exp OR 'wyoming'/exp |
| #2 | 'practice guideline'/exp OR 'practice guideline' OR 'physician'/exp OR 'physician' OR 'providers' OR 'clinician' OR 'clinicians' OR 'physician assistant'/exp OR 'physician assistant' OR 'nurse'/exp OR 'nurse' OR 'nurse practitioner'/exp OR 'nurse practitioner' OR ‘Academic detailing’ OR ‘continuing education’ OR ‘audit and feedback’ OR reminder OR opinion leader OR incentive OR training OR multifaceted OR office-based OR organizational OR practice patterns OR guideline adherence OR clinic OR clinics OR facility OR facilities OR hospital OR hospitals OR office OR offices OR practice OR practices OR primary care |
| #1 | 'observational studies'/exp OR 'observational studies' OR 'comparative studies'/exp OR 'comparative studies' OR 'randomized controlled trial'/exp OR 'randomized controlled trial' OR 'cohort analysis'/exp OR 'cohort analysis' OR 'randomized' OR 'cohort' OR 'intervention' OR 'intervention studies'/exp OR 'intervention studies' |

| **Search** | **29 March 2106 Web of Science query (k=1244)** |
| --- | --- |
| **#10** | **#7 OR #8 OR #9** |
| #9 | #1 AND #3 AND #4 AND #5 |
| #8 | #1 AND #4 AND #5 |
| #7 | (#1 AND #2 AND #3 AND #4 AND #5) **OR** (#3 AND #4 AND #5 AND #6) |
| #6 | migrant OR immigrant OR foreigners OR foreign-born OR refugee OR “high risk population” OR overseas OR chinese OR mexican OR guatemalan OR vietnamese OR haitian OR philippines OR china OR mexico OR guatemala OR haiti OR vietnam OR “viet nam” OR filipino |
| #5 | screening OR diagnosis OR diagnostic OR screen* OR test OR testing OR uptake OR recruit OR recruitment OR linked OR linkage |
| #4 | tuberculosis OR tb OR ltbi OR hepatitis OR hepatitis OR hbv OR hcv |
| #3 | “united states” OR alabama OR alaska OR arizona OR arkansas OR california OR colorado OR connecticut OR delaware OR florida OR georgia OR hawaii OR idaho OR illinois OR indiana OR iowa OR kansas OR kentucky OR louisiana OR maine OR maryland OR massachusetts OR michigan OR minnesota OR mississippi OR missouri OR montana OR nebraska OR nevada OR “new Hampshire” OR “new jersey” OR “new mexico” OR “new York” OR “north Carolina” OR “north Dakota” OR ohio OR oklahoma OR oregon OR pennsylvania OR “rhode island” OR “south Carolina” OR “south Dakota” OR tennessee OR texas OR utah OR vermont OR virginia OR washington OR “west Virginia” OR wisconsin OR wyoming |
| #2 | “practice guideline” OR physician OR physicians OR provider OR providers OR clinician OR clinicians OR “physician assistant” OR “physician assistants” OR nurse OR nurses OR nurse practitioner OR nurse practitioners OR "Academic detailing” OR continuing education OR “audit and feedback” OR reminder* OR opinion leader* OR incentive* OR training OR multifaceted OR office-based OR organizationalOR Physician's Practice Patterns[mh] OR practice patterns OR guideline adherence[mh] OR clinic* OR facility OR facilities OR hospital* OR office* OR practice* OR primary care[mh] OR primary care |
| #1 | observational OR comparative OR randomized controlled trial OR random* OR cohort OR cohorts OR intervention OR evaluation OR compared OR trial |

| **Search** | **29 March 2016 Cochrane Central Register query (k=468)** |
| --- | --- |
| **#10** | **#7 OR #8 OR #9** |
| #9 | #1 AND #3 AND #4 AND #5 |
| #8 | #1 AND #4 AND #5 |
| #7 | (#1 AND #2 AND #3 AND #4 AND #5) **OR** (#3 AND #4 AND #5 AND #6) |
| #6 | migrant OR immigrant OR foreigners OR foreign-born OR refugee OR “high risk population” OR overseas OR chinese OR mexican OR guatemalan OR vietnamese OR haitian OR philippines OR china OR mexico OR guatemala OR haiti OR vietnam OR “viet nam” OR filipino |
| #5 | screening OR diagnosis OR diagnostic OR screen* OR test OR testing OR uptake OR recruit OR recruitment OR linked OR linkage |
| #4 | tuberculosis OR tb OR ltbi OR hepatitis OR hepatitis OR hbv OR hcv |
| #3 | “united states” OR alabama OR alaska OR arizona OR arkansas OR california OR colorado OR connecticut OR delaware OR florida OR georgia OR hawaii OR idaho OR illinois OR indiana OR iowa OR kansas OR kentucky OR louisiana OR maine OR maryland OR massachusetts OR michigan OR minnesota OR mississippi OR missouri OR montana OR nebraska OR nevada OR “new Hampshire” OR “new jersey” OR “new mexico” OR “new York” OR “north Carolina” OR “north Dakota” OR ohio OR oklahoma OR oregon OR pennsylvania OR “rhode island” OR “south Carolina” OR “south Dakota” OR tennessee OR texas OR utah OR vermont OR virginia OR washington OR “west Virginia” OR wisconsin OR wyoming |
| #2 | “practice guideline” OR physician OR physicians OR provider OR providers OR clinician OR clinicians OR “physician assistant” OR “physician assistants” OR nurse OR nurses OR nurse practitioner OR nurse practitioners OR "Academic detailing” OR continuing education OR “audit and feedback” OR reminder* OR opinion leader* OR incentive* OR training OR multifaceted OR office-based OR organizational OR Physician's Practice Patterns OR practice patterns OR guideline adherence OR clinic* OR facility OR facilities OR hospital* OR office* OR practice* OR primary care |
| #1 | observational OR comparative OR randomized controlled trial OR random* OR cohort OR cohorts OR intervention OR evaluation OR compared OR trial |

**Updated Search Strategy**

**Our updated searches were conducted on 17 December 2018. Total records retrieved: 571. After removing 27 duplicate records, we screened 544 unique records obtained through updated database searches.**

| **Search** | **17 December 2018 PubMed query (k=34)** |
| --- | --- |
| **#9** | **#8 AND ("2016/03/29"[Date ­ Publication] : "3000"[Date ­ Publication])** |
| #8 | #7 AND Filters: Humans |
| #7 | (#1 AND #2 AND #3 AND #4 AND #5 AND #6) |
| #6 | Migrant*[tiab] OR immigrant*[tiab] OR foreigners[tiab] OR foreign-born[tiab] OR refugee*[tiab] OR high risk population*[tiab] OR overseas[tiab] OR chinese[tiab] OR mexican[tiab] OR guatemalan[tiab] OR vietnamese[tiab] OR haitian[tiab] OR philippines[tiab] OR china[tiab] OR mexico[tiab] OR guatemala[tiab] OR haiti[tiab] OR vietnam[tiab] OR “viet nam”[tiab] OR Filipino[tiab] OR Emigrants and Immigrants[mh] OR (“high-risk”[tiab] AND countr*[tiab]) OR ethnic groups[MeSH] OR language[tiab] |
| #5 | Mass screening[mh] OR screening[tiab] OR diagnosis[mh] OR diagnosis[tiab] OR diagnostic[tiab] OR screen*[tiab] OR test*[ti] OR uptake[tiab] OR recruit*[tiab] OR linked[tiab] OR linkage*[tiab] OR patient compliance[MeSH] |
| #4 | tuberculosis[mh] OR tuberculosis[tiab] OR tb[tiab] |
| #3 | “united states”[mh] OR “united states”[tiab] OR alabama[tiab] OR alaska[tiab] OR arizona[tiab] OR arkansas[tiab] OR california[tiab] OR colorado[tiab] OR connecticut[tiab] OR delaware[tiab] OR florida[tiab] OR georgia[tiab] OR hawaii[tiab] OR idaho[tiab] OR illinois[tiab] OR indiana[tiab] OR iowa[tiab] OR kansas[tiab] OR kentucky[tiab] OR louisiana[tiab] OR maine[tiab] OR maryland[tiab] OR massachusetts[tiab] OR michigan[tiab] OR minnesota[tiab] OR mississippi[tiab] OR missouri[tiab] OR montana[tiab] OR nebraska[tiab] OR nevada[tiab] OR “new Hampshire”[tiab] OR “new jersey”[tiab] OR “new mexico”[tiab] OR “new York”[tiab] OR “north Carolina”[tiab] OR “north Dakota”[tiab] OR ohio[tiab] OR oklahoma[tiab] OR oregon[tiab] OR pennsylvania[tiab] OR “rhode island”[tiab] OR “south Carolina”[tiab] OR “south Dakota”[tiab] OR tennessee[tiab] OR texas[tiab] OR utah[tiab] OR vermont[tiab] OR virginia[tiab] OR washington[tiab] OR “west Virginia”[tiab] OR wisconsin[tiab] OR Wyoming[tiab] OR "United States"[Grant Support] |
| #2 | practice guideline*[tiab] OR physicians[mh] OR physician*[tiab] OR provider[tiab] OR providers[tiab] OR clinician[tiab] OR clinicians[tiab] OR “physician assistants”[mh] OR physician assistant*[tiab] OR nurse[mh] OR nurse*[tiab] OR nurse practitioners[mh] OR nurse practitioner*[tiab] OR "Academic detailing”[tiab] OR continuing education[tiab] OR “audit and feedback”[tiab] OR reminder*[tiab] OR opinion leader*[tiab] OR incentive*[tiab] OR training[tiab] OR multifaceted[tiab] OR office-based[tiab] OR organizational[tiab]OR Physician's Practice Patterns[mh] OR practice patterns[tiab] OR guideline adherence[mh] OR clinic*[tiab] OR facility OR facilities[tiab] OR [tiab] OR hospital*[tiab] OR office*[tiab] OR practice*[tiab] OR primary care[mh] OR primary care[tiab] OR “performance improvement”[tiab] OR community health centers[mesh] OR (“public health”[tiab] AND program[tiab]) |
| #1 | observational[tiab] OR comparative[tiab] OR randomized controlled trial[pt] OR randomized controlled trial[tiab] OR random*[tiab] OR cohort studies[mh] OR cohort*[tiab] OR intervention[tiab] OR evaluation[tiab] OR compared[tiab] OR trial[tiab] OR assess*[tiab] OR “intervention group”[tiab] |

| **Search** | **17 December 2018 Embase query (k=129)** |
| --- | --- |
| **#9** | **#8 AND [29-3-2016]/sd NOT [19-12-2018]/sd** |
| #8 | #10 AND limit: Humans |
| #7 | (#1 AND #2 AND #3 AND #4 AND #5 AND #6) |
| #6 | 'migrant'/exp OR 'immigrant'/exp OR 'foreigners' OR 'foreign-born' OR 'refugee'/exp OR 'high risk population'/exp OR overseas OR 'chinese'/exp OR 'mexican'/exp OR 'guatemalan'/exp OR 'vietnamese'/exp OR 'haitian'/exp OR 'philippines'/exp OR 'china'/exp OR 'mexico'/exp OR 'guatemala'/exp OR 'haiti'/exp OR 'vietnam'/exp OR 'viet nam'/exp OR 'filipino':ab,ti |
| #5 | 'mass screening'/exp OR 'screening'/exp OR 'diagnosis'/exp OR 'diagnostic' OR 'screen':ti OR ‘test’:ti OR ‘testing’:ti OR uptake OR recruit OR recruitment OR linked OR linkage |
| #4 | 'tuberculosis'/exp/mj OR 'tuberculosis' OR 'tb'/exp/mj OR 'tb' OR ltbi |
| #3 | 'united states'/exp OR 'alabama'/exp OR 'alaska'/exp OR 'arizona'/exp OR 'arkansas'/exp OR 'california'/exp OR 'colorado'/exp OR 'connecticut'/exp OR 'delaware'/exp OR 'florida'/exp OR 'georgia' OR 'hawaii'/exp OR 'idaho'/exp OR 'illinois'/exp OR 'indiana'/exp OR 'iowa'/exp OR 'kansas'/exp OR 'kentucky'/exp OR 'louisiana'/exp OR 'maine'/exp OR 'maryland'/exp OR 'massachusetts'/exp OR 'michigan'/exp OR 'minnesota'/exp OR 'mississippi'/exp OR 'missouri'/exp OR 'montana'/exp OR 'nebraska'/exp OR 'nevada'/exp OR 'new hampshire'/exp OR 'new jersey'/exp OR 'new mexico'/exp OR 'new york'/exp OR 'north carolina'/exp OR 'north dakota'/exp OR 'ohio'/exp OR 'oklahoma'/exp OR 'oregon'/exp OR 'pennsylvania'/exp OR 'rhode island'/exp OR 'south carolina'/exp OR 'south dakota'/exp OR 'tennessee'/exp OR 'texas'/exp OR 'utah'/exp OR 'vermont'/exp OR 'virginia'/exp OR 'washington'/exp OR 'west virginia'/exp OR 'wisconsin'/exp OR 'wyoming'/exp |
| #2 | 'practice guideline'/exp OR 'practice guideline' OR 'physician'/exp OR 'physician' OR 'providers' OR 'clinician' OR 'clinicians' OR 'physician assistant'/exp OR 'physician assistant' OR 'nurse'/exp OR 'nurse' OR 'nurse practitioner'/exp OR 'nurse practitioner' OR ‘Academic detailing’ OR ‘continuing education’ OR ‘audit and feedback’ OR reminder OR opinion leader OR incentive OR training OR multifaceted OR office-based OR organizational OR practice patterns OR guideline adherence OR clinic OR clinics OR facility OR facilities OR hospital OR hospitals OR office OR offices OR practice OR practices OR primary care |
| #1 | 'observational studies'/exp OR 'observational studies' OR 'comparative studies'/exp OR 'comparative studies' OR 'randomized controlled trial'/exp OR 'randomized controlled trial' OR 'cohort analysis'/exp OR 'cohort analysis' OR 'randomized' OR 'cohort' OR 'intervention' OR 'intervention studies'/exp OR 'intervention studies' OR assess OR assessment OR ‘intervention group’ |

| **Search** | **17 December 2018 Web of Science query (k=56)** |
| --- | --- |
| **#8** | **#7 Refined by: PUBLICATION YEARS: ( 2018 OR 2017 OR 2016 )** |
| #7 | (#1 AND #2 AND #3 AND #4 AND #5 AND #6) |
| #6 | migrant OR immigrant OR foreigners OR foreign-born OR refugee OR “high risk population” OR overseas OR chinese OR mexican OR guatemalan OR vietnamese OR haitian OR philippines OR china OR mexico OR guatemala OR haiti OR vietnam OR “viet nam” OR filipino OR (“high-risk” AND countr*) OR “ethnic group*” OR language |
| #5 | screening OR diagnosis OR diagnostic OR screen* OR test OR testing OR uptake OR recruit OR recruitment OR linked OR linkage OR “patient compliance” |
| #4 | tuberculosis OR tb OR ltbi |
| #3 | “united states” OR alabama OR alaska OR arizona OR arkansas OR california OR colorado OR connecticut OR delaware OR florida OR georgia OR hawaii OR idaho OR illinois OR indiana OR iowa OR kansas OR kentucky OR louisiana OR maine OR maryland OR massachusetts OR michigan OR minnesota OR mississippi OR missouri OR montana OR nebraska OR nevada OR “new Hampshire” OR “new jersey” OR “new mexico” OR “new York” OR “north Carolina” OR “north Dakota” OR ohio OR oklahoma OR oregon OR pennsylvania OR “rhode island” OR “south Carolina” OR “south Dakota” OR tennessee OR texas OR utah OR vermont OR virginia OR washington OR “west Virginia” OR wisconsin OR wyoming |
| #2 | “practice guideline” OR physician OR physicians OR provider OR providers OR clinician OR clinicians OR “physician assistant” OR “physician assistants” OR nurse OR nurses OR nurse practitioner OR nurse practitioners OR "Academic detailing” OR continuing education OR “audit and feedback” OR reminder* OR opinion leader* OR incentive* OR training OR multifaceted OR office-based OR organizationalOR Physician's Practice Patterns[mh] OR practice patterns OR guideline adherence[mh] OR clinic* OR facility OR facilities OR hospital* OR office* OR practice* OR primary care[mh] OR primary care OR “performance improvement” OR “health cent*” OR (“public health” AND program) |
| #1 | observational OR comparative OR randomized controlled trial OR random* OR cohort OR cohorts OR intervention OR evaluation OR compared OR trial OR assess* OR “intervention group” |

| **Search** | **17 December 2018 Cochrane Central Register query (k=352)** |
| --- | --- |
| **#8** | **#7 with Cochrane Library publication date from Mar 2016 to present** |
| #7 | (#1 AND #2 AND #3 AND #4 AND #5 AND #6) |
| #6 | migrant OR immigrant OR foreigners OR foreign-born OR refugee OR “high risk population” OR overseas OR chinese OR mexican OR guatemalan OR vietnamese OR haitian OR philippines OR china OR mexico OR guatemala OR haiti OR vietnam OR “viet nam” OR filipino OR (“high-risk” AND countr*) OR “ethnic group*” OR language |
| #5 | screening OR diagnosis OR diagnostic OR screen* OR test OR testing OR uptake OR recruit OR recruitment OR linked OR linkage OR “patient compliance” |
| #4 | tuberculosis OR tb OR ltbi |
| #3 | “united states” OR alabama OR alaska OR arizona OR arkansas OR california OR colorado OR connecticut OR delaware OR florida OR georgia OR hawaii OR idaho OR illinois OR indiana OR iowa OR kansas OR kentucky OR louisiana OR maine OR maryland OR massachusetts OR michigan OR minnesota OR mississippi OR missouri OR montana OR nebraska OR nevada OR “new Hampshire” OR “new jersey” OR “new mexico” OR “new York” OR “north Carolina” OR “north Dakota” OR ohio OR oklahoma OR oregon OR pennsylvania OR “rhode island” OR “south Carolina” OR “south Dakota” OR tennessee OR texas OR utah OR vermont OR virginia OR washington OR “west Virginia” OR wisconsin OR wyoming |
| #2 | "practice guideline" OR physician OR physicians OR provider OR providers OR clinician OR clinicians OR "physician assistant" OR "physician assistants" OR nurse OR nurses OR nurse practitioner OR nurse practitioners OR "Academic detailing" OR continuing education OR "audit and feedback" OR reminder* OR opinion leader* OR incentive* OR training OR multifaceted OR office-based OR organizational OR "Physician's Practice Patterns" OR "practice patterns" OR "guideline adherence" OR clinic* OR facility OR facilities OR hospital* OR office* OR practice* OR "primary care" OR "performance improvement" OR "health cent*" OR ("public health" AND program) |
| #1 | observational OR comparative OR randomized controlled trial OR random* OR cohort OR cohorts OR intervention OR evaluation OR compared OR trial OR assess* OR “intervention group” |
