# Supplementary material for: Healthcare facility-based strategies to improve tuberculosis testing and linkage to care in non-U.S.-born population in the United States: A systematic review
Source: PLoS One. 2019 Sep 30;14(9):e0223077. doi: 10.1371/journal.pone.0223077 (PMC6768470; doi:10.1371/journal.pone.0223077)
Supplement: S2 Appendix — (DOCX) [file pone.0223077.s003.docx]

# S2 Appendix: Articles screened for inclusion at the full text level

Table of Contents

[S2. Appendix B: Articles screened for inclusion at the full text level 1](#_Toc9864700)

[Included Studies (k=7) 1](#_Toc9864701)

[Excluded: No Comparator (k=20) 3](#_Toc9864702)

[Excluded: Not healthcare facility-based (K=12) 5](#_Toc9864703)

[Excluded: Doesn’t report outcome of interest (k=9) 7](#_Toc9864704)

[Excluded: Hepatitis Study (k=4)* 8](#_Toc9864705)

[Excluded: No intervention (k=4) 9](#_Toc9864706)

[Excluded: Sample identified during immigration process (k=4) 9](#_Toc9864707)

[Excluded: Ineligible target population (k=3) 10](#_Toc9864708)

[Excluded: Conducted outside of the U.S. (k=2) 11](#_Toc9864709)

[Excluded: Pediatric study (k=1) 11](#_Toc9864710)

[Excluded: Insufficient data (k=1) 11](#_Toc9864711)

[Excluded: Review paper (k=2) 11](#_Toc9864712)

## Included Studies (k=7)

Anand A, Wagner C, Kong SS, Griffith E, Harimtepathip P, Baker KK, et al. Improving Screening for Latent Tuberculosis Infection in a Student-run Free Clinic. Cureus. 2018;10(4):e2488.

Kempker JA, Pasquel FJ, Castejon MS, Acosta A, Zaragoza-Macias E, Ilksoy N, et al. Quality improvement of tuberculosis screening in foreign-born patients. Journal of immigrant and minority health / Center for Minority Public Health. 2012;14(1):1-5

Leng JC, Changrani J, Gany FM. Language discordance and testing for latent tuberculosis infection among recent Asian and Latino immigrants. Journal of community health [Internet]. 2011; 36(2):[228-30 pp.]. Available from: http://onlinelibrary.wiley.com/o/cochrane/clcentral/articles/875/CN-00787875/frame.html.

Schultz JS, Floyd E, Guitierrez C, Manning W, Newton TP, Frank A, et al. Improving latent tuberculosis screening in a combined internal medicine and pediatrics residency outpatient clinic at denver health-a resident designed and implemented quality improvement project. Journal of General Internal Medicine. 2018;33(2):240-1.

Steele AW, Eisert S, Davidson A, Sandison T, Lyons P, Garrett N, et al. Using computerized clinical decision support for latent tuberculosis infection screening. American journal of preventive medicine. 2005;28(3):281-4.

Tanke ED, Leirer VO. Automated Telephone Reminders in Tuberculosis Care. Medical Care. 1994;32(4):380-9.

Tanke ED, Martinez CM, Leirer VO. Use of automated reminders for tuberculin skin test return. American journal of preventive medicine [Internet]. 1997; 13(3):[189-92 pp.]. Available from: http://onlinelibrary.wiley.com/o/cochrane/clcentral/articles/476/CN-00140476/frame.html

## Excluded: No Comparator (k=20)

Al-Tayyib A, Ginnett L, Edel M, Weise J, Thrun M. PCSI in practice: Fully integrated screening for hepatitis C virus in an STD clinic. Sexually Transmitted Diseases. 2014;41:S127.

Barnett S, Little L. TB Education and Targeted Testing of Garfield County, Colorado, WIC Clients. TB Notes. 2006(2):21-3.

Cain KP, Garman KN, Laserson KF, Ferrousier-Davis OP, Miranda AG, Wells CD, et al. Moving toward Tuberculosis Elimination Implementation of Statewide Targeted Tuberculin Testing in Tennessee. American Journal of Respiratory and Critical Care Medicine. 2012;186(3):273-9.

Chandrasekar E, Kaur R, Song S, Kim KE. A comparison of effectiveness of hepatitis B screening and linkage to care among foreign-born populations in clinical and nonclinical settings. Journal of Multidisciplinary Healthcare. 2015;8:1-9.

Davidow AL, Katz D, Ghosh S, Blumberg H, Tamhane A, Sevilla A, et al. Preventing Infectious Pulmonary Tuberculosis Among Foreign-Born Residents of the United States. American Journal of Public Health. 2015;105(9):E81-E8.

Davidow AL, Sevilla A, Katz D, Reves R, Epidemiologic Studies Consortium TB. Prevention of tuberculosis in older foreign-born residents of the US. American Journal of Respiratory and Critical Care Medicine. 2010;181(1).

D'Lugoff MI, Jones W, Kub J, Glass N, Thompson D, Brinkley-Laughon S, et al. Tuberculosis screening in an at-risk immigrant Hispanic population in Baltimore city: an academic health center/local health department partnership. Journal of cultural diversity. 2002;9(3):79-85.

Duchen D, Boyd AT, Annamalai A. Screening and Treatment Rates for Latent Tuberculosis Among Newly-Arrived Refugees in an Urban Facility in Connecticut. Connecticut medicine. 2017;81(5):291-8.

Gjerdingen DK, Lor V. Hepatitis B status of Hmong patients. The Journal of the American Board of Family Practice / American Board of Family Practice. 1997;10(5):322-8.

Haley C. A Statewide Targeted Tuberculin Testing Program In Tennessee. TB Notes. 2004(1):7-9.

Harrowe D. 230505: Diagnosis and treatment of latent tuberculosis infection: Working with community health clinics and undertaking a separate project with foreign-born physicians. American Public Health Association (APHA), 138nd Annual Meeting & Expo; Nov 6-10, 2010; Denver, CO 2010.

Jonas MM, Schiff ER, O'Sullivan MJ, de Medina M, Reddy KR, Jeffers LJ, et al. Failure of Centers for Disease Control criteria to identify hepatitis B infection in a large municipal obstetrical population. Annals of internal medicine. 1987;107(3):335-7.

Kallman JB, Tran S, Arsalla A, Haddad D, Stepanova M, Fang Y, et al. Vietnamese community screening for hepatitis B virus and hepatitis C virus. Journal of viral hepatitis. 2011;18(1):70-6.

Kallman JB, Tran SV, Arsalla A, Haddad D, Stepanova M, Yun F, et al. Screening for Hepatitis B (Hbv) and Hepatitis C (Hcv) in a Vietnamese Community of Northern Virginia. Hepatology. 2009;50(4):656A-A.

Morano JP, Walton MR, Zelenev A, Bruce RD, Altice FL. Latent tuberculosis infection: screening and treatment in an urban setting. J Community Health. 2013;38(5):941-50.

Morano JP, Zelenev A, Walton MR, Bruce RD, Altice FL. Latent Tuberculosis Infection Screening in Foreign-Born Populations: A Successful Mobile Clinic Outreach Model. American Journal of Public Health. 2014;104(8):1508-15.

Morisky DE, Malotte CK, Ebin V, Davidson P, Cabrera D, Trout PT, et al. Behavioral interventions for the control of tuberculosis among adolescents. Public health reports (Washington, DC : 1974) [Internet]. 2001; 116(6):[568-74 pp.]. Available from: <http://onlinelibrary.wiley.com/o/cochrane/clcentral/articles/942/CN-00390942/frame.html>.

Norton D. Tuberculosis screening for international students. Journal of American college health : J of ACH. 2000;48(4):187-9.

Nuzzo JB, Golub JE, Chaulk P, Shah M. Postarrival Tuberculosis Screening of High-Risk Immigrants at a Local Health Department. American Journal of Public Health. 2015;105(7):1432-8.

Perumalswami PV, Factor SH, Kapelusznik L, Friedman SL, Pan CQ, Chang C, et al. Hepatitis Outreach Network: A practical strategy for hepatitis screening with linkage to care in foreign-born communities. Journal of Hepatology. 2013;58(5):890-7.

Tin K, Perumalswami P, Vanderhoff AM, Carmody E, Dieterich D, Culpepper-Morgan J, et al. Hepatitis B in west African-born persons living in New York City: Is linkage to care enough? American Journal of Gastroenterology. 2015;110:S967.

## Excluded: Not healthcare facility-based (K=12)

Chang ET, Sue E, Zola J, So SK. 3 For Life: a model pilot program to prevent hepatitis B virus infection and liver cancer in Asian and Pacific Islander Americans. American journal of health promotion : AJHP. 2009;23(3):176-81.

Chen MS, Fang DM, Stewart SL, Ly MY, Lee S, Dang JHT, et al. Increasing Hepatitis B Screening for Hmong Adults: Results from a Randomized Controlled Community-Based Study. Cancer Epidemiology Biomarkers & Prevention. 2013;22(5):782-91.

Dang JHT, Chen MS. Increasing Hepatitis B Testing and Linkage to Care of Foreign-Born Asians, Sacramento, California, 2012–2013. Public Health Reports 2016;131(S2).

Gany FM, Trinh-Shevrin C, Changrani J. Drive-by readings: a creative strategy for tuberculosis control among immigrants. American journal of public health. 2005;95(1):117-9.

Jackson AE, Jeffers A, Sirotin N, Kunins H, Osinaga A. Retention and screening rates of immigrant patients in the south bronx. Journal of General Internal Medicine. 2011;26:S313-S4.

Juon HS, Lee S, Strong C, Rimal R, Kirk GD, Bowie J. Effect of a Liver Cancer Education Program on Hepatitis B Screening Among Asian Americans in the Baltimore Washington Metropolitan Area, 2009-2010. Preventing Chronic Disease. 2014;11.

Malotte CK, Rhodes F, Mais KE. Tuberculosis screening and compliance with return for skin test reading among active drug users. American journal of public health [Internet]. 1998; 88(5):[792-6 pp.]. Available from: <http://onlinelibrary.wiley.com/o/cochrane/clcentral/articles/016/CN-00684016/frame.html>.

O'Donnell MR, Chamblee S, von Reyn CF, Marsh BJ, Moreland JD, Narita M, et al. Sustained reduction in tuberculosis incidence following a community-based participatory intervention. Public health action. 2012;2(1):23-6.

Poss JE, Rangel R. A tuberculosis screening and treatment program for migrant farmworker families. Journal of health care for the poor and underserved. 1997;8(2):133-40.

Taylor VM, Hislop TG, Tu SP, Teh C, Acorda E, Yip MP, et al. Evaluation of a hepatitis B lay health worker intervention for Chinese Americans and Canadians. Journal of community health [Internet]. 2009; 34(3):[165-72 pp.]. Available from: <http://onlinelibrary.wiley.com/o/cochrane/clcentral/articles/625/CN-00700625/frame.html>.

Taylor VM, Yasui Y, Burke N, Choe JH, Acorda E, Jackson JC. Hepatitis B knowledge and testing among Vietnamese-American women. Ethnicity & disease. 2005;15(4):761-7.

Wurtele SK, Galanos AN, Roberts MC. Increasing return compliance in a tuberculosis detection drive. J Behav Med. 1980;3(3):311-8.

## Excluded: Doesn’t report outcome of interest (k=9)

Carabez R. 306716: Curbing the Hepatitis B Epidemic in Asian American Communities: Engaging Local Hospitals. American Public Health Association (APHA), 142nd Annual Meeting & Expo; November 15-19, 2014; New Orleans, LA 2014.

Goldberg SV, Wallace J, Jackson JC, Chaulk CP, Nolan CM. Cultural case management of latent tuberculosis infection. The international journal of tuberculosis and lung disease : the official journal of the International Union against Tuberculosis and Lung Disease. 2004;8(1):76-82.

Hass MR. Health seeking and patient adherence: Tuberculosis screening and Latino immigrants. [Dissertation]. Ann Arbor, MI: University of California, Irvine; 1993.

Macaraig M, Alexander M. New York City Bureau of TB Control Restructures Clinic Services. TB Notes. 2010(4):9-11.

Madison CM. A pharmacist lead latent tuberculosis infection clinic in a county health department. Pharmacotherapy. 2015;35(11):e220.

McDonald CJ, Hui SL, Smith DM, Tierney WM, Cohen SJ, Weinberger M, et al. Reminders to physicians from an introspective computer medical record. A two-year randomized trial. Annals of internal medicine. 1984;100(1):130-8.

Morano JP, Zelenev A, Lombard A, Marcus R, Gibson BA, Altice FL. Strategies for hepatitis C testing and linkage to care for vulnerable populations: point-of-care and standard HCV testing in a mobile medical clinic. J Community Health. 2014;39(5):922-34.

Nolan CM. Community-wide implementation of targeted testing for and treatment of latent tuberculosis infection. Clinical infectious diseases: an official publication of the Infectious Diseases Society of America. 1999;29(4):880-7.

Shah M, DiPietro D, Greenbaum A, Ketemepi S, Martins-Evora M, Marsiglia V, et al. Programmatic Impact of QuantiFERON-TB Gold In-Tube Implementation on Latent Tuberculosis Diagnosis and Treatment in a Public Health Clinic. Plos One. 2012;7(5).

## Excluded: Hepatitis Study (k=4)*

** These articles met all other inclusion criteria and if we hadn’t found a sufficient number of TB articles, they would have been included in the systematic review. Hepatitis articles categorized under other exclusion justifications still wouldn’t have met inclusion criteria if hepatitis articles were included but all other criteria were left the same.*

Drainoni ML, Litwin AH, Smith BD, Koppelman E, McKee MD, Christiansen CL, et al. Effectiveness of a risk screener in identifying hepatitis C virus in primary care. Hepatology. 2011;54:591A.

Hsu L, Bowlus CL, Stewart SL, Nguyen TT, Dang J, Chan B, et al. Electronic messages increase hepatitis B screening in at-risk Asian-American patients: A randomized, controlled trial. Digestive Diseases and Sciences [Internet]. 2013; 58(3):[807-14 pp.]. Available from: <http://onlinelibrary.wiley.com/o/cochrane/clcentral/articles/491/CN-00907491/frame.html>.

Litwin AH, Smith BD, Drainoni ML, McKee D, Gifford AL, Koppelman E, et al. Primary care-based interventions are associated with increases in hepatitis C virus testing for patients at risk. Digestive and liver disease: official journal of the Italian Society of Gastroenterology and the Italian Association for the Study of the Liver. 2012;44(6):497-503.

Waltermaurer E, Wilson G. Leveraging Electronic Health Records and Specific Demographic Data to Screen for Hepatitis B in a Community Health Center. American Public Health Association 140th Annual Meeting: Prevention and Wellness Across the Life Span; October 29, 2012; San Francisco, CA 2012.

## Excluded: No intervention (k=4)

Chuang J, Fitzgerald SM, Berger Y, Perumalswami P, Sarpel U. HBV and HCC Screening Practices in Immigrant-rich Neighborhoods of New York City. Annals of Surgical Oncology. 2015;22:S105-S.

Giama NH, Shire A, Shaleh HM, Mohamed EA, Roberts LR. Community-wide outreach and screening to reduce hepatitis B and hepatitis C disparities among Somali immigrants in Minnesota. Hepatology. 2014;60:960A-A.

Oh PKM. The demography of tuberculosis in California in a time of transition: In search of empirical evidence to guide public health agencies’ efforts to target tuberculosis screening in immigrant communities [Dissertation]. Ann Arbor, MI: University of California, Berkeley; 2015.

Reddy D, Walker J, White LF, Brandeis GH, Russell ML, Horsburgh CR, et al. Latent Tuberculosis Infection Testing Practices in Long-Term Care Facilities, Boston, Massachusetts. Journal of the American Geriatrics Society. 2017;65(6):1145-51.

## Excluded: Sample identified during immigration process (k=4)

Carter KL, Gabrellas AD, Shah S, Garland JM. Improved latent tuberculosis therapy completion rates in refugee patients through use of a clinical pharmacist. International Journal of Tuberculosis and Lung Disease. 2017;21(4):432-7.

Posey DL, Naughton MP, Willacy EA, Russell M, Olson CK, Godwin CM, et al. Implementation of new TB screening requirements for U.S.-bound immigrants and refugees - 2007-2014. MMWR Morbidity and mortality weekly report. 2014;63(11):234-6.

Subedi P, Drezner KA, Dogbey MC, Newbern EC, Yun K, Scott KC, et al. Evaluation of latent tuberculous infection and treatment completion for refugees in Philadelphia, PA, 2010-2012. International Journal of Tuberculosis and Lung Disease. 2015;19(5):565-9.

Waldorf B, Gill C, Crosby SS. Assessing adherence to accepted national guidelines for immigrant and refugee screening and vaccines in an urban primary care practice: a retrospective chart review. Journal of immigrant and minority health / Center for Minority Public Health. 2014;16(5):839-45.

## Excluded: Ineligible target population (k=3)

Merchant RC, Baird JR, Liu T, Taylor LE, Montague BT, Nirenberg TD. Brief intervention to increase emergency department uptake of combined rapid human immunodeficiency virus and hepatitis C screening among a drug misusing population. Academic emergency medicine : official journal of the Society for Academic Emergency Medicine [Internet]. 2014; 21(7):[752-67 pp.]. Available from: <http://onlinelibrary.wiley.com/o/cochrane/clcentral/articles/415/CN-01002415/frame.html>.

Schluter WW, Ralston DL, Delaney RJ, Sauaia A, Dunn TR. Increasing influenza and pneumococcal vaccination and tuberculosis screening among residents of Colorado long-term care facilities. Evaluation and the Health Professions. 1999;22(4):466-83.

Sidlow R, Msaouel P. Improving Hepatitis C Virus Screening Rates in Primary Care: A Targeted Intervention Using the Electronic Health Record. Journal for Healthcare Quality. 2015;37(5):319-

## Excluded: Conducted outside of the U.S. (k=2)

Caballeria L, Pera G, Bernad J, Canut S, Navarro E, Bruguera M. Strategies for the detection of hepatitis C viral infection in the general population. Rev Clin Esp. 2014;214(5):242-6.

Griffiths C, Sturdy P, Brewin P, Bothamley G, Eldridge S, Martineau A, et al. Educational outreach to promote screening for tuberculosis in primary care: a cluster randomised controlled trial. Lancet (London, England). 2007;369(9572):1528-34.

## Excluded: Pediatric study (k=1)

Bordley WC, Margolis PA, Stuart J, Lannon C, Keyes L. Improving preventive service delivery through office systems. Pediatrics. 2001;108(3):E41.

## Excluded: Insufficient data (k=1)

Carlson EK, Miller TL, Katz DJ, Vemulapalli A, Fa A, Rohrback AC, et al. Practical economic evaluation in the local setting: A web-based tool to guide latent tuberculosis infection (LTBI) screening and treatment. American Journal of Respiratory and Critical Care Medicine. 2014;189.

## Excluded: Review paper (k=2)

Arditi C, Rège‐Walther M, Durieux P, Burnand B. Computer‐generated reminders delivered on paper to healthcare professionals: effects on professional practice and healthcare outcomes. Cochrane Database of Systematic Reviews. 2017(7).

Kahwati LC, Feltner C, Halpern M, Woodell CL, Boland E, Amick HR, et al. Screening for Latent Tuberculosis Infection in Adults: An Evidence Review for the U.S. Preventive Services Task Force. Research Triangle Park, North Carolina: RTI International-–University of North Carolina Evidence-based Practice Center, 2016 Evidence Synthesis, No.142 Contract No.: HHSA-290-2012-00015-I, Task Order No. 4.
